# Quality of life and depression among chronic kidney disease patients: a tertiary care center cross-sectional study

**DOI:** 10.3389/fneph.2025.1600296

**Published:** 2025-10-02

**Authors:** Rana A. Nablawi, Lama S. Alghamdi, Adnan E. Alshaikh, Abdullah M. Alagha, Nada T. Alharbi, Abdulah H. Ali, Hassan A. Khafaji, Essam W. E Zarei, Nabil A. Alzahrani

**Affiliations:** ^1^ Department of Internal Medicine, Faculty of Medicine, King Abdulaziz University, Jeddah, Saudi Arabia; ^2^ Faculty of Medicine, University of Jeddah, Jeddah, Saudi Arabia; ^3^ Faculty of Medicine, King Abdulaziz University, Jeddah, Saudi Arabia; ^4^ Faculty of Medicine, Taibah University, Medina, Saudi Arabia

**Keywords:** chronic kidney disease, depression, quality of life, patient health questionnaire-9, 12-item short form health survey, physical component summary score, mental component summary score

## Abstract

**Background:**

Chronic kidney disease (CKD) is a severe health condition that involves a decline in kidney function, leading to high mortality rates in Saudi Arabia and globally. It often coexists with chronic non-communicable conditions such as hypertension and diabetes. As CKD progresses, patients experience psychological distress, anxiety, and depression, which can negatively impact their health and quality of life. This can lead to reduced treatment adherence, increased mortality, and poor quality of life.

**Objective:**

This study sought to assess the prevalence of depression among CKD patients, investigate how quality of life (QoL) and depression vary across CKD stages, and examine the relationship between depression and QoL at different disease stages. This study was conducted at a tertiary care center in Jeddah, Saudi Arabia.

**Methods:**

This cross-sectional research, conducted in Jeddah, Saudi Arabia, from February to May 2024, included 95 CKD patients who met the CKD diagnostic criteria as confirmed by a nephrologist. Pregnant women, dialysis patients, and patients under the age of 18 were excluded from the research. Patients’ contact information was gathered from electronic medical records at King Abdulaziz University Hospital (KAUH), and consent was sought over the phone. Depression was assessed in non-dialysis CKD patients using the Patient Health Questionnaire (PHQ-9), and health-related quality of life (HRQoL) was assessed using the 12-Item Short-Form Health Survey (SF-12) score. Demographic information, previous medical comorbidities, and estimated glomerular filtration rate (eGFR) were also considered. The 2012 Kidney Disease: Improving Global Outcomes (KDIGO) CKD classification was used to classify patients into stages. The research sought to give a full evaluation of patients’ mental and physical health.

**Results:**

A total of 95 patients were included in this study, with a predominance of male gender (58.9%) and those who were aged 60 years and above (50.5%). Most patients were non-smokers (78.9%), and 45.3% were classified as non-obese patients. Comorbidities were widespread among these patients, especially hypertension (82.1%) and diabetes (74.7%). Regarding severity level measured by PHQ-9, the median score was 12.0, 28.4% of the patients were classified as having moderate depression, and the correlation between depression and physical activity (PCS12) and mental health (MCS12) was significantly negative. Multiple linear regression analysis showed that depression was significantly associated with lower physical and mental capacity scores, alongside older age and female gender.

**Conclusion:**

This study emphasized the substantial impact of depressive symptoms among obese patients, highlighting the interplay between mental health and chronic physical conditions. Our findings suggest that specific risk factors such as fatigue, chronic illness, including hypertension, and prior mental health history are associated with increased vulnerability to depression. These insights underscore the importance of integrating routine mental health screening and individualized intervention strategies into patient care, particularly for those with comorbid conditions. Future research is needed to further explore the causal relationships and to inform more effective, targeted public health.

## Introduction

Chronic kidney disease (CKD) is a progressive condition characterized by a gradual decline in kidney function, classified into distinct stages based on the severity. Globally, CKD is an escalating health burden, ranking among the major causes of mortality. In Saudi Arabia, it is the third leading cause of death (1, 2). A notional laboratory-based study from 2015 to 2022 reported an overall CKD prevalence of 4.76%, with mostly stage 3 (3.5%) (3, 4). As kidney function deteriorates, patients often face complex physical, emotional, and social challenges, leading to increased risk of co-existing comorbid conditions, including diabetes mellitus and cardiovascular disease, which contribute to functional decline and negatively impact quality of life (QoL) (5, 6). While the physical consequences of CKD are well understood, the psychological aspect, particularly depression, remains underrecognized, especially among patients not yet receiving dialysis.

Depression is a prevalent mental health illness in CKD patients. A study conducted in 2022 confirmed that depression is more common among CKD patients, especially those experiencing end-stage renal disease (ESRD) (7). Furthermore, a Saudi-based study found that 24.6% of CKD patients undergoing hemodialysis had depression, with none receiving antidepressant treatment (8, 9). Despite this, limited attention has been given to depressive symptoms and QoL in non-dialysis CKD populations.

Quality of life is a multidimensional concept encompassing physical, emotional, and social well-being. It is profoundly affected by CKD progression. Depression has been shown to correlate strongly with reduced QoL, and both factors have been linked to poor clinical outcomes, including lower treatment adherence, increased hospitalization, and elevated mortality rates (10–12).

Recent evidence highlights the importance of assessing health-related quality of life (HRQoL) and using validated tools such as the Patient Health Questionnaire (PHQ-9) to evaluate depressive symptoms in chronic disease populations (13). These assessments provide critical insight into patient functioning and guide decision-making. CKD patients, especially those on dialysis, often report psychological and financial burden that further impacts their overall well-being. Enhancing patient education and integrating psychological support into CKD care can improve self-management and promote adherence to treatment plans (14).

While several studies have assessed depression and QoL in dialysis populations, the prevalence of depression and impact on health-related QoL remain underexplored in non-dialysis populations receiving conservative treatment for CKD (15). Divergent findings regarding the effects of age on QoL have been reported, with some studies negating its significance while others identifying age as the foremost determinant of QoL (16). Additionally, concerning related decision-making in the therapy plan for CKD, considering the choice of the better outcomes, in terms of the quality of life, survival percentage, and accompanying treatment options, between conservative therapy and dialysis (17), some studies have shown that conservative kidney management is a highly valuable replacement for traditional therapies for CKD patients (18). Six discriminations in CKD have also been investigated, with some studies finding that women have poorer outcomes than men (16).

Therefore, this study aimed to assess the prevalence of depression and evaluate its relationship with quality of life among non-dialysis CKD patients. By examining these outcomes across different CKD stages, we sought to better understand the psychological needs of this population and establish a supportive care plan that enhances patient quality of life and patient outcomes.

## Materials and methods

### Study design and settings

This was a cross-sectional study conducted at a tertiary care center in Jeddah, Saudi Arabia, from February to May 2024. The objectives were to determine the prevalence of depression in patients with CKD, investigate and assess QoL and depression across the CKD stages, and ascertain the relationship between depression and QoL among CKD patients with their disease stages.

### Study participants and ethical considerations

A total of 95 patients were diagnosed with CKD by a nephrologist according to the 2012 Kidney Disease: Improving Global Outcomes (KDIGO) guidelines. CKD was defined as abnormalities of kidney structure or function, present for more than 3 months, with health implications, including either an estimated glomerular filtration rate (eGFR) <60 mL/min/1.73 m^2^ or markers of kidney damage (such as albuminuria) for ≥3 months (19, 20).

Patients on dialysis (either hemodialysis or peritoneal dialysis) were excluded to avoid confounding, as this group has a known higher prevalence of depression due to treatment-related burdens. Additionally, pregnant women and individuals younger than 18 years were excluded. While prior psychiatric illness was not part of the exclusion criteria, we aimed to capture the current prevalence and severity of depression within non-dialysis CKD populations. However, all participants were assessed for depressive symptoms using the PHQ-9 at the time of enrollment. The study received approval from the Institutional Review Board (IRB) of KAUH (Reference Number 241-24).

### Data collection

The data collection process started in March 2024, and all patients’ contact details were obtained from the internal medicine department’s electronic medical records (EMRs) at KAUH. All CKD patients who visited KAUH from January 2017 to December 2022 were retrospectively collected. This temporal gap between the patient records and the conduction of the study was due to a major update to the hospital’s data system, which enabled better access and review of the records. To mitigate this, the dataset was carefully reviewed to ensure that the variables and the outcomes remain consistent with current standards of care and diagnostic criteria.

We called each patient and acquired their verbal consent for the survey due to the retrospective nature of the study. The research team interviewed those who provided consent. This approach was approved by the IRB as ethically acceptable and aligned with the type of study.

All interviewers have received proper standardized training regarding the data collection process, which included instructions on how to administer each part of the questionnaires consistently and how to communicate naturally with the patients to reduce interviewer bias. We used a data collection sheet using Google Forms, including the PHQ-9 as a screening instrument for depression and the 12-item Short-Form Health Survey as an assessment tool for quality of life in non-dialysis CKD patients, both of which were validated and translated into Arabic to be used on Arabic populations (21, 22). Clinical and sensitive variables such as drug use history were primarily extracted through patient self-report due to confidentiality issues.

Depression was categorized into five levels: minimal (0–4), mild (5–9), moderate (10–14), moderately severe (15–19), and severe (20–27) (23).

The physical component summary (PCS) and the mental component summary (MCS) are two summary scores of the 12-Item Short-Form Health Survey (SF-12). All scales were assessed using the conventional approach from the SF-12 scoring manual. Each score runs from 0 to 100, with higher scores indicating greater health-related quality of life (HRQoL). A score of 50 or less on the PCS has been proposed as a cut-off to determine a physical condition; however, a score of 42 or less on the MCS may indicate clinical depression (24).

Demographic variables included age, gender, body mass index (BMI), smoking status, and alcohol or illegal drug use. Past medical comorbidities, such as diabetes mellitus, hypertension, heart failure, stroke, and dyslipidemia, were recorded. While illicit drug use is not a conventional comorbidity in CKD classification, it was included as a behavioral health variable due to its potential impact on mental health and treatment adherence.

The eGFR was calculated using the Chronic Kidney Disease Epidemiology Collaboration equation (CKD-EPI) creatinine-based formula (20) based on the most recent serum creatinine measurement before the survey. CKD staging followed the 2012 KDIGO CKD classification: stages G1 to G3a (eGFR ≥ 45 mL/min/1.73 m^2^) were grouped as early-stage CKD, while stages G3b to G5 (eGFR < 45 mL/min/1.73 m^2^) were considered as late-stage CKD (25).

### Statistical analysis

All statistical analyses were performed using the RStudio software (R version 4.3.1). Descriptive statistics were used to summarize the demographic and clinical characteristics of the patients. Continuous variables were reported as median [interquartile range (IQR)] and mean ± SD, while categorical variables were presented as frequencies and percentages. Generalized linear regression analysis was conducted for univariable and multivariable analyses to identify significant factors associated with the PHQ-9, PCS12, and MCS12 scores. The multivariable models included the significantly associated variables from the univariable analysis to be used as independent variables. The depression scores (PHQ-9) and quality of life (PCS12 and MCS12) were used as dependent variables, each in a separate model. A p-value <0.05 was considered statistically significant, and no formal adjustments for multiple comparisons were applied, as the analyses were exploratory and intended to identify potential associations to guide future research. There were no missing data.

## Results

### Demographic and clinical characteristics of patients

**Figure 1:**
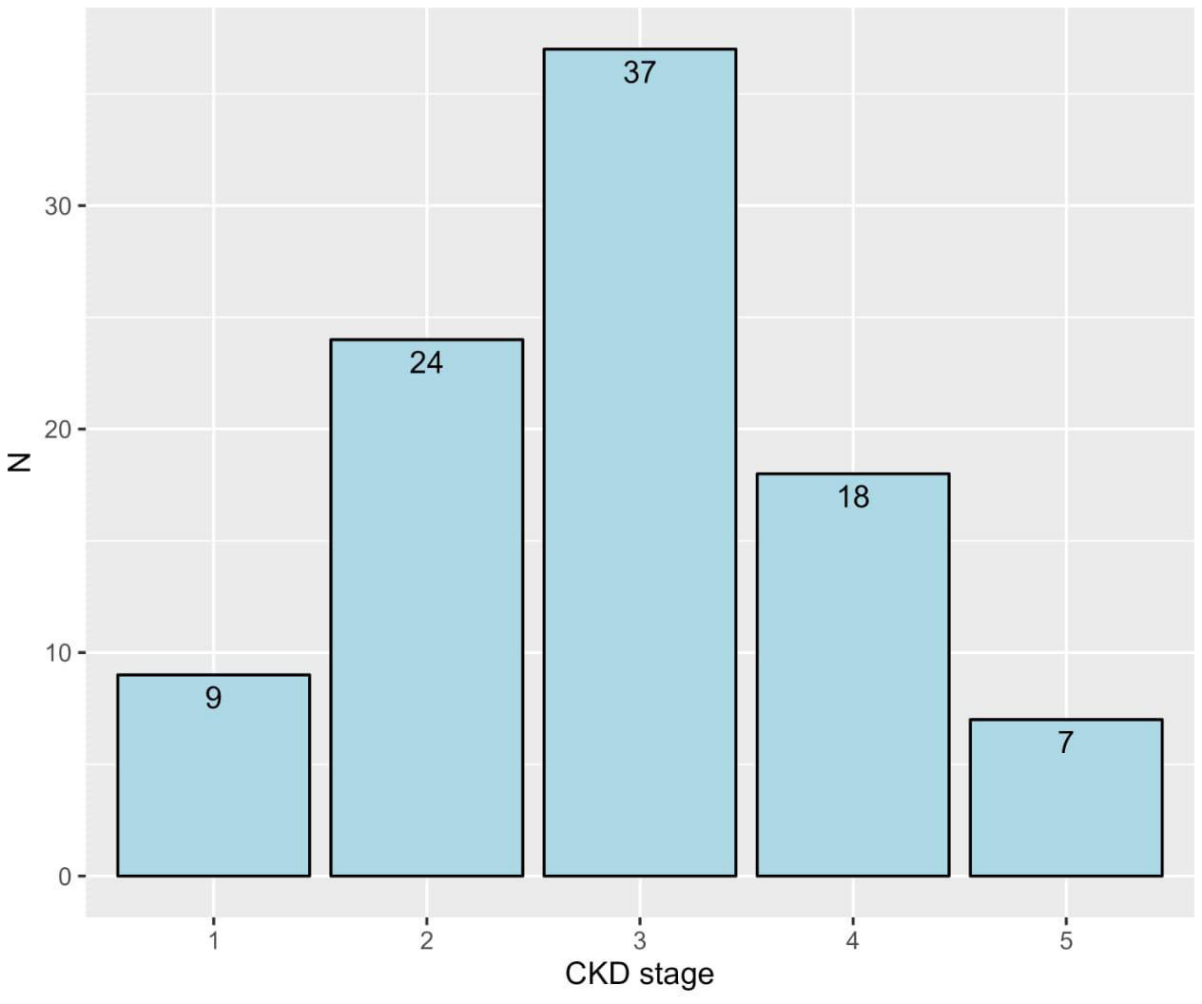
The frequencies of patients with different CKD stages. CKD, chronic kidney disease.

The study included a total of 95 CKD patients, with the majority being 60 years or older (50.5%) and predominantly male (58.9%). Most patients were non-smokers (78.9%), with a smaller proportion being current smokers (4.2%) or ex-smokers (16.8%). In terms of BMI, a significant number of patients were classified as obese (54.7%), followed by overweight (26.3%). The prevalence of comorbid conditions was notably high, with diabetes (74.7%), hypertension (82.1%), and dyslipidemia (42.1%) being the most common. Most patients were in the early stages of CKD (stages 1 to 3, 73.7%), with the remaining in the late stages (stages 4 and 5, 26.3%; **Table 1**). More details about the frequencies of CKD stages are depicted in **Figure 1**.

**Table 1:** Demographic and clinical characteristics of patients.

Characteristic	Description
Age	
18 to <30	7 (7.4%)
30 to <45	9 (9.5%)
45 to <60	31 (32.6%)
60 or more	48 (50.5%)
Gender	
Male	56 (58.9%)
Female	39 (41.1%)
Smoking status	
Current smoker	4 (4.2%)
Ex-smoker	16 (16.8%)
Non-smoker	75 (78.9%)
BMI	
Underweight	1 (1.1%)
Healthy	17 (17.9%)
Overweight	25 (26.3%)
Obese	52 (54.7%)
Comorbidities	
Diabetes	71 (74.7%)
Hypertension	78 (82.1%)
Heart failure	3 (3.2%)
Stroke	8 (8.4%)
Dyslipidemia	40 (42.1%)
Alcohol or illicit drug	2 (2.1%)
CKD stage	
Early	70 (73.7%)
Late	25 (26.3%) n
n (%)	

BMI, body mass index; CKD, chronic kidney disease.

### Description of depression and quality of life

**Figure 2:**
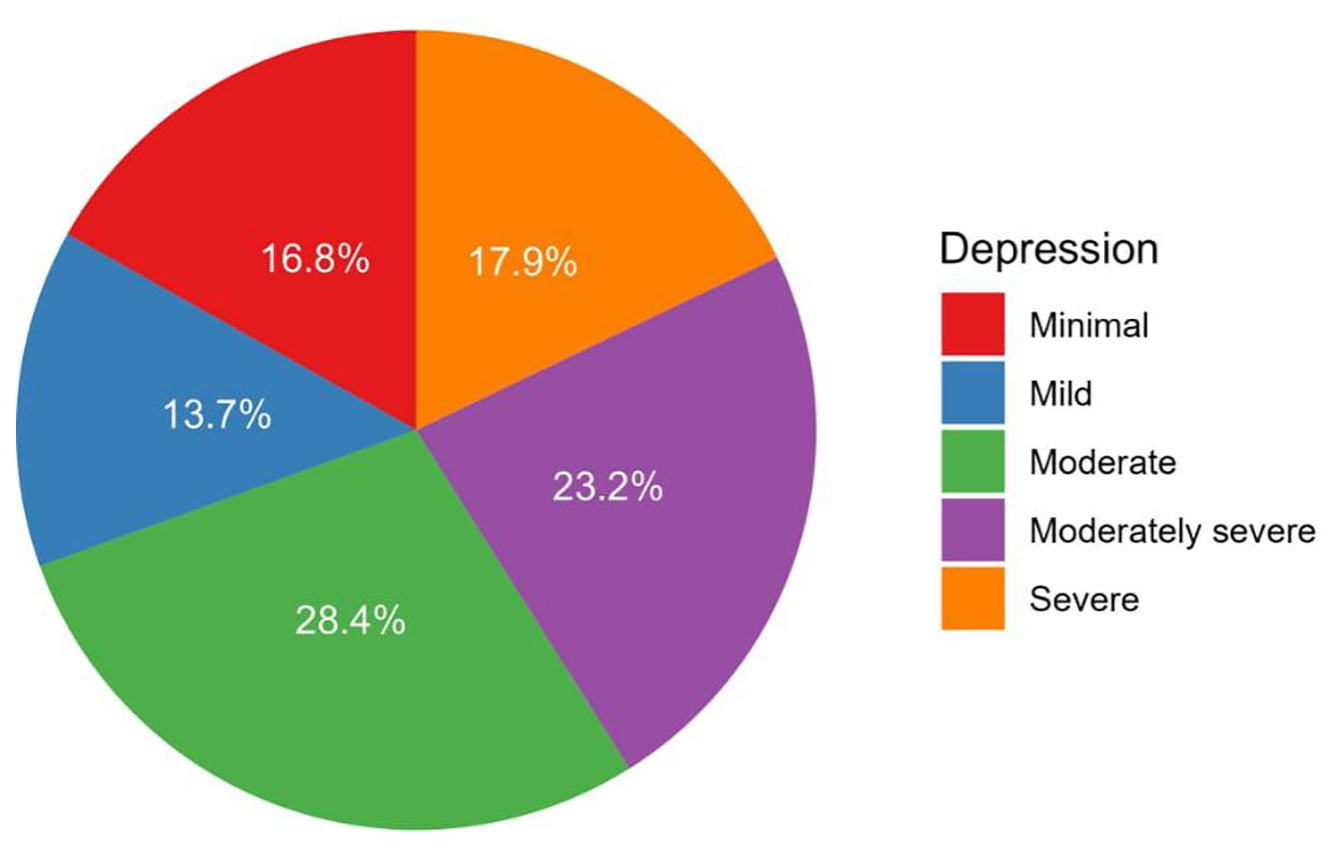
A pie chart depicting the proportion of depression categories.

The median PHQ-9 score for depression was 12.0 (IQR: 3.0–16.0), ranging from 0.0 to 27.0 (**Table 2**). Depression severity among patients was categorized as none–minimal (16.8%), mild (13.7%), moderate (28.4%), moderately severe (23.2%), and severe (17.9%, **Figure 2**).

**Table 2:** Description of the numerical scores.

Characteristic	Median (IQR)	Mean ± SD	Min–Max
PHQ-9 score (depression)	12.0 (3.0–16.0)	10.7 ± 7.1	0.0–27.0
PCS12	44.2 (34.6–50.2)	42.7 ± 10.3	21.1–60.7
MCS12	39.6 (32.5–50.1)	41.2 ± 11.6	16.0–59.7

PHQ, Patient Health Questionnaire; PCS, physical component summary; MCS, mental component summary; IQR, interquartile range; SD, standard deviation.

The Physical Component Summary (PCS12) had a median score of 44.2 (IQR: 34.6–50.2), whereas the Mental Component Summary (MCS12) had a median score of 39.6 (IQR: 32.5–50.1, **Table 2**).

### Factors and predictors of the depression score

**Figure 3:**
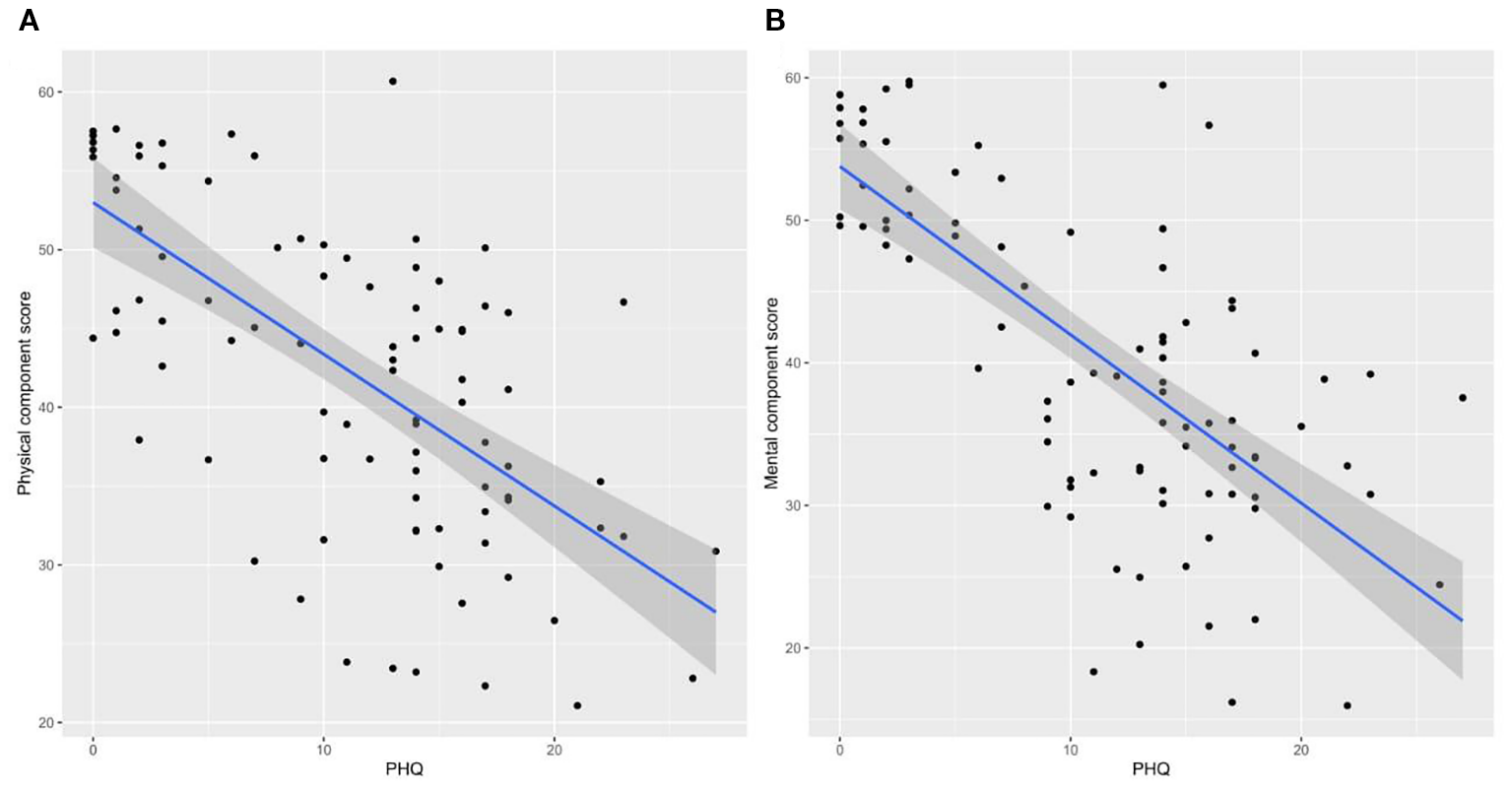
Bivariate correlations between the depression score and physical component score **(A)** and between the depression score and mental component score **(B)**.

In the univariable analysis, significant factors of higher PHQ-9 scores included age 60 or more (beta = 6.7, 95% CI: 1.2 to 12, p = 0.019) and female gender (beta = 3.0, 95% CI: 0.15 to 5.8, p = 0.042). Multivariable regression confirmed age 60 or more (beta = 6.66, 95% CI: 1.24 to 12.1, p = 0.018) and female gender (beta = 3.03, 95% CI: 0.20 to 5.85, p = 0.038) as significant predictors. Other factors such as smoking status, BMI, comorbidities, illicit drug use, and CKD stage were not significant predictors (**Table 3**). The bivariate correlations indicated a negative correlation between the PHQ-9 score and both the PCS12 (**Figure 3A**) and the MCS12 (**Figure 3B**).

**Table 3:** Factors and predictors of the PHQ-9 score as indicators of depression.

Characteristic	Univariable analysis			Multivariable regression		
	Beta	95% CI	p-Value	Beta	95% CI	p-Value
Age						
18 to <30	Reference	Reference		Reference	Reference	
30 to <45	6.5	−0.40, 13	0.068	7.10	0.32, 13.9	0.043
45 to <60	5.5	−0.21, 11	0.062	5.62	0.02, 11.2	0.052
60 or more	6.7	1.2, 12	0.019	6.66	1.24, 12.1	0.018
Gender						
Male	Reference	Reference		Reference	Reference	
Female	3.0	0.15, 5.8	0.042	3.03	0.20, 5.85	0.038
Smoking status						
Current smoker	Reference	Reference				
Ex-smoker	3.2	−4.5, 11	0.416			
Non-smoker	5.9	−1.2, 13	0.105			
BMI						
Underweight/healthy	Reference	Reference				
Overweight	1.0	−3.3, 5.4	0.639			
Obese	1.2	−2.6, 5.1	0.527			
Diabetes						
No	Reference	Reference				
Yes	2.8	−0.44, 6.0	0.094			
Hypertension						
No	Reference	Reference				
Yes	−1.2	−4.9, 2.5	0.534			
Heart failure						
No	Reference	Reference				
Yes	3.4	−4.7, 12	0.410			
Stroke						
No	Reference	Reference				
Yes	0.77	−4.4, 5.9	0.771			
Dyslipidemia						
No	Reference	Reference				
Yes	0.13	−2.8, 3.0	0.929			
Alcohol or illicit drug						
No	Reference	Reference				
Yes	−5.3	−15, 4.6	0.298			
CKD stage						
Early	Reference	Reference				
Late	0.06	−3.2, 3.3	0.970			

CI, confidence interval; BMI, body mass index; CKD, chronic kidney disease; PHQ, Patient Health Questionnaire.

### Factors and predictors of the physical component score

The univariable analysis identified age (beta = −12, 95% CI: −21 to −1.7, p = 0.024 for those aged 30 to <45 years; beta = −9.1, 95% CI: −17 to −0.97, p = 0.031 for those aged 45 to <60 years; and beta = −12, 95% CI: −20 to −3.8, p = 0.004 for those aged 60 or more), diabetes (beta = −5.8, 95% CI: −10 to −1.2, p = 0.015), and the PHQ-9 score (beta = −0.96, 95% CI: −1.2 to −0.74, p < 0.001) as significant factors associated with lower PCS scores. However, multivariable regression showed that the PHQ-9 score for depression (beta = −0.89, 95% CI: −1.12 to −0.66, p < 0.001) was the only significant independent predictor (**Table 4**).

**Table 4:** Factors and predictors of the physical component score as indicators of physical quality of life.

Characteristic	Univariable analysis			Multivariable regression		
	Beta	95% CI	p-Value	Beta	95% CI	p-Value
Age						
18 to <30	Reference	Reference		Reference	Reference	
30 to <45	−12	−21, −1.7	0.024	−5.28	−13.0, 2.44	0.184
45 to <60	−9.1	−17, −0.97	0.031	−2.72	−9.40, 3.96	0.427
60 or more	−12	−20, −3.8	0.004	−4.13	−10.7, 2.44	0.221
Gender						
Male	Reference	Reference				
Female	−2.4	−6.6, 1.8	0.266			
Smoking status						
Current smoker	Reference	Reference				
Ex-smoker	−7.6	−19, 3.6	0.187			
Non-smoker	−9.1	−19, 1.2	0.087			
BMI						
Underweight/healthy	Reference	Reference				
Overweight	0.61	−5.7, 6.9	0.850			
Obese	0.52	−5.0, 6.1	0.856			
Diabetes						
No	Reference	Reference		Reference	Reference	
Yes	−5.8	−10, −1.2	0.015	−2.92	−6.81, 0.96	0.144
Hypertension						
No	Reference	Reference				
Yes	2.5	−2.9, 7.8	0.374			
Heart failure						
No	Reference	Reference				
Yes	−3.5	−15, 8.3	0.562			
Stroke						
No	Reference	Reference				
Yes	−4.2	−12, 3.2	0.266			
Dyslipidemia						
No	Reference	Reference				
Yes	−2.0	−6.2, 2.1	0.341			
Alcohol or illicit drug						
No	Reference	Reference				
Yes	7.4	−6.9, 22	0.313			
CKD stage						
Early	Reference	Reference				
Late	2.1	−2.6, 6.8	0.374			
PHQ-9 score (depression)	−0.96	−1.2, −0.74	<0.001	−0.89	−1.12, −0.66	<0.001

CI, confidence interval; BMI, body mass index; CKD, chronic kidney disease; PHQ, Patient Health Questionnaire.

### Factors and predictors of the mental component score

The univariable analysis identified the PHQ-9 score for depression (beta = −1.18, 95% CI: −1.41 to −0.95, p < 0.001) as the sole significant factor for lower MCS scores (**Table 5**). Therefore, conducting a multivariable model for MCS12 prediction was not feasible.

**Table 5:** Factors and predictors of the mental component score as indicators of mental quality of life.

Characteristic	Univariable analysis		
	Beta	95% CI	p-Value
Age			
18 to <30	Reference	Reference	
30 to <45	−5.4	−17, 5.9	0.354
45 to <60	−2.3	−12, 7.1	0.635
60 or more	−7.9	−17, 1.1	0.090
Gender			
Male	Reference	Reference	
Female	−4.2	−8.9, 0.48	0.082
Smoking status			
Current smoker	Reference	Reference	
Ex-smoker	−2.1	−15, 10	0.747
Non-smoker	−7.8	−19, 3.7	0.189
BMI			
Underweight/healthy	Reference	Reference	
Overweight	−2.6	−9.7, 4.5	0.472
Obese	−0.47	−6.7, 5.8	0.884
Diabetes			
No	Reference	Reference	
Yes	−1.4	−6.8, 3.9	0.600
Hypertension			
No	Reference	Reference	
Yes	−0.65	−6.8, 5.5	0.835
Heart failure			
No	Reference	Reference	
Yes	−6.8	−20, 6.5	0.320
Stroke			
No	Reference	Reference	
Yes	0.57	−7.9, 9.0	0.894
Dyslipidemia			
No	Reference	Reference	
Yes	−0.56	−5.3, 4.2	0.818
Alcohol or illicit drug			
No	Reference	Reference	
Yes	−2.1	−18, 14	0.799
CKD stage			
Early	Reference	Reference	
Late	−4.5	−9.8, 0.71	0.094
PHQ-9 score (depression)	−1.18	−1.41, −0.95	<0.001

CI, confidence interval; BMI, body mass index; CKD, chronic kidney disease; PHQ, Patient Health Questionnaire.

## Discussion

In this study, we aimed to assess the prevalence of depression and quality of life among CKD patients. We investigated 95 CKD patients, most of whom were older, aged 60 years or older (50.5%), and men (58.9%). This is in line with previous research in the field, demonstrating the potential vulnerability of these subgroups to both increased depression risk and decreased quality of life (26). Despite being conducted in a single but tertiary center, we believe that the sample is reasonably representative of a broader CKD population due to the inclusion of different age groups, genders, CKD stages, and comorbidities in Saudi Arabia.

The prevalence of comorbid diseases such as hypertension (82.1%), diabetes (74.7%), and dyslipidemia (42.1%) among CKD patients in this study was notable and aligns with prior findings. For instance, a US study showed that CKD patients had a similar prevalence of diabetes and hypertension to our study, which suggests the interrelationship between these diseases and the development of CKD (27). However, these comorbidities not only increase the progression of CKD but also affect its management and, therefore, affect the patient’s quality of life (11, 28).

Moreover, our study observed that most of our patients were in early stages of CKD, in which 73.7% were in stages 1–3, with a lower percentage of 26.3% in the late stages, stages 4 and 5. These observations align with those of the International Society of Nephrology, which indicates that CKD is usually diagnosed in its early stages (29). A possible explanation for this finding is the presence of screening efforts and increased awareness about CKD, which agrees with a Chinese study (30). Regardless of these efforts, the progression of CKD to late stages is still a major concern. This demonstrates the need for more effective interventions and more investigations into the factors that could affect the progression of the disease.

Additionally, this study showed that the median PHQ-9 score for depression among CKD patients was 12.0, which ranges from 0.0 to 27.0. We also found that the largest percentage of the patients suffered from moderate depression (28.4%), a less significant percentage suffered from moderately severe depression (23.2%), and 17.9% suffered from severe depression. These findings coincide with those of the Palmer et al. study, which indicates a significant occurrence of depression among CKD patients (31). Moreover, the disparity in the depression severity in this study illustrates the complex interplay of psychological factors that influence CKD patients, pointing out the importance of routine mental health assessment and a directed management plan. A possible explanation for the high severity scores of the depressive symptoms that are experienced by CKD patients is that these patients face significant emotional distress, which negatively impacts their quality of life and may affect their physical health.

Furthermore, when investigating the variables of higher PHQ-9 scores, we found that age 60 and older and female gender were significant. However, smoking status, BMI, comorbidities, illicit drug usage, and CKD stage were not significant predictors. Although smokers in our sample appeared to have lower PHQ-9 scores, the association was not statistically significant and should not be interpreted as protective. These findings are consistent with those of a Jordanian study, which indicated the possible susceptibility of these subgroups to develop more severe depressive symptoms (32). However, a possible explanation for the lack of significance for other factors is that the psychological burden and the effects on depression of CKD exceed the disease’s physical severity. As for this issue, addressing the mental and psychological needs of all CKD patients is required, regardless of their CKD stages or the presence of other comorbidities.

Moreover, several studies have found a strong association between depression and chronic conditions such as diabetes and hypertension; our findings did not suggest these associations. This may be related to sample size limitations and the presence of unmeasured confounding factors. Moreover, it is possible that the psychological effect of CKD may overshadow the effect of individual comorbidities on depression severity.

Also, our study found that there is a relationship between PHQ-9 score and poorer physical component summary (PCS12) and mental component summary (MCS12). This indicates that depression symptoms have a major impact on both the mental and physical health of CKD patients. This aligned with other research, which demonstrated that depression coexisting with CKD may have important additional adverse effects on functional status and physical health (31). Also, our study’s univariable analysis showed that age and diabetes are associated with lower PCS scores. However, in our multivariable analysis, only depressive symptoms remained a significant predictor of PCS scores. This indicates that depressive symptoms have a more significant effect on physical health. Other research revealed that depression was associated with subjective well-being, physical well-being, and environmental circumstances (33). Moreover, our study’s univariable analysis showed that the PHQ-9 score was a significant factor for mental component summary (MCS 12) scores.

Our study has several limitations. First, the sample size of only 95 patients restricts the generalizability of the results, which is one of the study’s many drawbacks, and this could also affect the subgroup analysis. Second, self-reported quality of life (MCS 12 and PCS 12) and depression (PHQ-9) assessments may have increased bias response. Due to another aspect that affects the accuracy of the findings, the patient may overreport or underreport depressive symptoms. Moreover, self-perception could be affected by temporary emotional conditions, which could affect the patient at the time of the interview. Third, patients’ sensitive variable details, such as illicit drug use, were based only on self-reported data collected from the patient. Data were not cross-validated with hospital records due to confidentiality issues. Lastly, uncontrollable factors that affect both depression and quality of life were not taken into consideration in this study, including socioeconomic status, cultural factors, medication adherence, and social support.

However, the study’s recruitment method, which relied on contact information from medical records and obtaining phone consent, may have introduced participation bias, as patients who were more ill, less motivated, or unreachable may be underrepresented. Additionally, response rates and refusal counts were not systematically recorded.

Finally, while our results support depression screening in CKD care, recommendations should be interpreted in the context of our single tertiary-center Saudi cohort. Broader application requires validation in multicenter and cross-cultural settings. In practice, screening could be integrated using brief tools like PHQ-9 during clinic visits. Positive screens should lead to structured referral pathways, staff training in symptom recognition, and inclusion of mental health professionals in nephrology teams. Further research is needed and should build on the limitations in our study to identify the complex relationship between CKD and the development of depression.

## Conclusion

The purpose of the study was to ascertain the prevalence of depression in individuals with CKD, investigate and evaluate depression and QoL with various stages of the disease, and ascertain the relationship between these variables in CKD patients. We discovered that more than half of the patients had moderately to moderately severe depression, and nearly three-quarters of the patients were in the early stages of CKD. Furthermore, in comparison to the SF-12 questionnaire summary score cut-off, the CKD patients in our study exhibited lower quality of life levels. The significant risk factors for increased depression were female gender and age 60 and older. There was a negative link between the patient’s quality of life and the degree of depression, while other variables, including smoking status, BMI, comorbidities, illicit drug use, and CKD stage, were also not significant predictors. Patients with diabetes, those over 30, and the PHQ-9 score of depression were all found to be significantly linked with decreased PCS scores. The only significant factor for lower MCS scores was the PHQ-9 score for depression, as this was a cross-sectional study; these relationships represent associations and do not imply causality.

Lastly, we recommend using a validated questionnaire like the PHQ-9 to screen all CKD patients for depression, particularly those over 60 and women. In addition, treating depression in CKD patients implies a multidisciplinary team approach. Additionally, we advise educating CKD patients specifically about the dangers of depression and low quality of life, as well as providing ongoing care and monitoring. To clarify the risk of depression and low quality of life in patients with non-dialysis chronic kidney disease, larger sample sizes must be used in future studies.

## Data Availability

The original contributions presented in the study are included in the article/supplementary material. Further inquiries can be directed to the corresponding author.
